# Polycyclic aromatic hydrocarbons content of food, water and vegetables and associated cancer risk assessment in Southern Nigeria

**DOI:** 10.1371/journal.pone.0306418

**Published:** 2024-07-23

**Authors:** Augusta Chinyere Nsonwu-Anyanwu, Mohamed Helal, Azza Khaked, Raymond Eworo, Chinyere Adanna Opara Usoro, Amany EL-Sikaily

**Affiliations:** 1 Department of Clinical Chemistry and Immunology, University of Calabar, Calabar, Nigeria; 2 National Institute of Oceanography and Fisheries, Cairo, Egypt; 3 Department of Biology, University of Southern Denmark, Odense, Denmark; 4 Biochemistry Department, College of Medicine, University of Hail, Hail, Saudi Arabia; Center for Research and Technology Transfer, VIET NAM

## Abstract

The polycyclic aromatic hydrocarbon content of water (four surface water, six underground water (borehole water), seven sachet water), barbecued food and their fresh equivalents (barbecued beef, fish, plantain, pork, yam, chicken, chevon, potato, corn), oil (three palm oil, nine vegetable oil), and fresh vegetable samples (water leaf, bitter leaf, cabbage, carrot, cucumber, pumpkin, garlic, ginger, green leaf, *Gnetum Africana*, onion, pepper) were determined by GC-MS analysis. The current study also determined the estimated lifetime cancer risk from ingesting polycyclic aromatic hydrocarbon-contaminated food. The polycyclic aromatic hydrocarbon content of water, oil, vegetable, and food samples were within the United States Environmental Protection Agency/World Health Organization safe limits. The naphthalene, benzo(b)fluoranthene, and benzo(k)fluoranthene levels in surface water were significantly higher than in borehole samples (*P* = 0.000, 0.047, 0.047). Vegetable oils had higher anthracene and chrysene compared to palm oil (*P* = 0.023 and 0.032). Significant variations were observed in levels of naphthalene, acenaphthylene, phenanthrene, benzo(b)fluoranthene, benzo(k)fluoranthene, benzo(a)pyrene, and dibenzo(a,h)anthracene among the barbecued and fresh food samples (*P* <0.05). Barbecued pork, potato, and corn had significantly higher naphthalene compared to their fresh equivalents (*P* = 0.002, 0.017, and <0.001). Consumption of barbecued food and surface water may be associated with higher exposure risk to polycyclic aromatic hydrocarbons which may predispose to increased cancer health risk. The current work explores in depth the concentration of polycyclic aromatic hydrocarbons in different dietary categories that pose direct risk to humans via direct consumption. These findings add knowledge to support future considerations for human health.

## Introduction

The pervasion of environmental pollution with PAHs represents a public health concern due to their documented toxic, mutagenic, and carcinogenic characteristics [1]. PAHs are ubiquitous organic contaminants consisting of two or more fused aromatic rings of carbon and hydrogen atoms. They are primarily produced through natural and anthropogenic activities. The main sources of PAHs include combustion or pyrolysis (incomplete burning) of wood, coal, oil, organic matter, industrial emissions, and other human activities such as motor vehicle exhaust, sewage discharge, tobacco, and barbecue smoke [2–5]. The intricate amalgam of PAHs generated through these processes tends to accumulate in the environment, thereby contaminating the air, water, and soil which ultimately integrate into the human food chain [2, 6]. Human exposure to PAHs has been linked with deleterious health consequences; their epoxide metabolites exhibit binding/modifying affinity for DNA and proteins leading to a mutagenic cascade and increasing cancer predisposition risk [1]. As evidenced by experimental in vivo and in vitro studies, the United State Environmental Protection Agency (USEPA) has declared 16 PAHs as priority pollutants and seven as carcinogenic by the International Agency for Research on Cancer [7]. PAH toxicity is dependent on the route of exposure, concentration level, duration, and PAH mixture composition to which the individual is exposed; a composite exposure of two or more PAHs is likely to be more carcinogenic than an individual PAH. Among different PAHs, Benzo(a)pyrene is the most extensively studied member [8].

Previous research indicated that the mean concentration of PAHs in leavy products in Southern Nigeria ranged from 532 to 2261 μg kg^−1^ [9]. PAH content in soil and vegetation products in Nigeria with average PAH concertation raging from 10 to 35 mg·kg^−1^ [10]. Another study evaluated the PAH content of smoked fish in Nigeria with the report of high PAH4 index in smoked fish samples and high cancer risk [11]. However, on the environmental and health levels there is a lack of information on the current concentration and carcinogenicity of PAHs in different dietary exposure routes (such as water, food, meat, and cooking oil)

On the environmental level, contamination of surface and underground water occurs via landfill leaching, petroleum spills, fossil fuel combustion, and improper disposal of industrial wastes [12]. Surface water is more vulnerable to PAH contamination than underground, rendering the consumption of surface water due to a lack of safety. Studies indicate that drinking water samples that have undergone prechlorination, aeration, and disinfection treatment procedures exhibit PAH levels five times lower than untreated water samples [13, 14].

On the health level, the major routes of exposure to PAHs include inhalation, ingestion, and dermal contact in both occupational and non-occupational contexts [2]. food constitutes the major route of exposure to PAHs for non-smokers and non-occupationally exposed individuals [15] with contamination commonly arising from air, soil, water, and food processing & cooking methods. Given the relatively low PAH content in raw food items, cooking methods such as grilling, smoking, and drying are the major contributors to PAH contamination, and consumption of these food items serves as a major means of PAH exposure [1]. The PAH content of food is dependent on the cooking recipe, time, fuel type, distance of food from the heat source, and food fat content [16]. Furthermore, the contamination of vegetables with PAH occurs via the gaseous deposition from the atmosphere and soil uptake. The extent of PAH content in vegetables is positively correlated with the leaf surface area, as larger surface areas facilitate greater absorption of PAHs from the atmosphere [17, 18]. Leafy vegetables such as spinach, white gourds, fenugreeks, chilis, ribbed gourds, and cauliflowers exhibit higher PAH content compared to underground ones such as potatoes, radishes, and turnips [19]. Naturally obtained vegetable oils are typically devoid of PAHs. However, oil seed drying, oil solvent extraction, and package material are possible PAH contamination processes for these oils. Refined oils typically exhibit lower levels of PAHs than crude oils [20–22].

Human exposure to unpredictable levels of PAHs in food, water, and vegetables often contributes to the development of systemic ailments [23–25]. The environmental risk assessment of different food items is an important study for the healthy life of human beings [1, 25]. Up to our knowledge, there has been no complete relevant available data on PAH levels in food and water from Calabar, Southern Nigeria in which environmental contamination and dietary habits are different from other areas.

An understanding of the PAH content of these food items habitually consumed in the locality will offer a comprehensive risk assessment of PAHs required to control PAH in food for a healthier and wealthier population. In this context, this study assessed the PAH content of surface and underground water samples, barbecued food and their fresh equivalents, vegetable oil, palm oil, fresh vegetables, and the estimated lifetime cancer risk (ELCR) accruing from ingestion of PAH-contaminated food in Calabar Southern Nigeria.

## Material and methods

### Study area and design

The research work was undertaken in Cross River State, located in the southern region of Nigeria at coordinates 5.045°N, and 8.030°E. The study focused on two local governmental areas, namely: Calabar Municipal and Calabar South, which collectively constitute the Calabar metropolis region. Calabar, the capital city of Cross River State, is spreading over an estimated area of 406 km^2^ with 371,022 population. The city is comprised of twenty-two wards, positioned between latitudes 4.015° and 5.0° N and longitudes 8.025° and 8.0° E. The indigenous Qua and Efik populations predominate in the region, as documented by the National Population Commission/National Bureau of Statistics in 2006. The majority of the population in the study area are civil servants while the main industry is the University of Calabar.

### Ethical consideration

A written informed consent was sought and obtained from all study participants before experiment enrollment. The ethical approval was obtained from the Cross River State Ministry of Health Research Ethics Committee (REC No. CRSMOH/RP/REC/2021/210). This study was carried out in accordance with the Ethical Principles for Medical Research involving human subjects as outlined in the Helsinki Declaration in 1975 and subsequent revisions.

### Sample collection

Different representative environmental and food samples were collected and included in the current study. A total of 24 water samples (four surface water (SWS), six underground water (borehole water (BWS)), seven sachet water, seven bottled water samples; twelve oil samples (three palm oil, nine vegetable oil); twelve vegetable samples (each of water leaf, bitter leaf, cabbage, carrot, cucumber, pumpkin, garlic, ginger, green leaf, *Gnetum Africana (Okazi)*, onion and pepper) and five food samples each contain barbecued beef, fish, plantain, pork, yam, chicken, chevon, potato and corn and their fresh equivalents respectively were collected from five major food joints located in Calabar, Southern Nigeria. All collected samples were sorted and ice transported to the laboratory and kept at 4 °C before digestion and processing.

#### Food samples

Five food samples of barbecued beef, fish, plantain, pork, yam, chicken, chevon, potato, and corn and their fresh equivalents respectively were collected from the Atimbo roundabout food outlet, Atekong junction food outlet, Ekpo Abasi roundabout food outlet, Goldie roundabout food outlet, and Calabar Road roundabout food outlet all located within the Calabar metropolis, Cross River State Nigeria. The selection of the food outlets was based on the patronage. The meat samples were taken from the thigh region of the animal’s body. All meat samples were kept at 4 °C before digestion and processing. One hundred grams of each food sample was collected, wrapped with foil paper, and stored at -20 °C until further PAH extraction and GC-MS analysis.

#### Water samples

Five hundred milliliters of each water sample were collected. Samples include four SWS, six borehole samples from public water hot spot services (BWS), seven bottled water samples, and seven sachet water samples purchased from local vendors for PAH extraction and GC-MS analysis. The four surface water samples were collected downstream of the Marina Resort River, Unical River, Akpabuyo River, and Anagtiga Stream which were designated as SWS1 to SWS4. The six borehole (underground) water samples were randomly collected, three from Calabar Municipal and three from Calabar South, and labeled BWS one to six. Water samples were collected by submerging a metal plastic-free container at a depth of 10–20 cm under the surface while borehole samples were collected directly from tap sources. All containers were rinsed at least three times with distilled water and the source water before sample collection. The collected water samples were transferred in ice boxes to the laboratory for the extraction and analysis of 16 priority PAHs.

#### Vegetable samples

Twelve vegetable samples (250g) each of water leaf, bitter leaf, cabbage, carrot, cucumber, pumpkin, garlic, ginger, green leaf, *Gnetum Africana (Okazi)*, onion, and pepper were procured from local wholesale markets to measure the levels of PAH exposure level of the population near market places. The vegetable samples were wrapped in aluminum foil and kept refrigerated at 4°C, until the time of analysis.

#### Vegetable oil

Twelve oil samples (one liter each) comprising three palm oil samples and nine different brands of vegetable oil samples habitually consumed were purchased from local markets. The vegetable oil samples were kept at room temperature and analyzed within one week.

### Laboratory methods

#### Sample preparation

Freshly collected vegetable samples were thoroughly washed with distilled water and air-dried. Barbecued and air-dried vegetable samples were oven-dried at 70°C to remove residual moisture. Dried samples were crushed with a pestle and mortar.

#### PAH extraction from vegetable, vegetable oil, and barbecued samples

As adopted from the previously established method [9], ten grams of each respective food sample and their fresh equivalents were extracted with 250 mL of methanol (HPLC grade, Sigma Aldrich), followed by a saponification step with 25 ml of 1M KOH solution. Upon saponification and to collect the PAHs dissolved compounds, samples were shake-extracted 3x with n-hexane (HPLC grade, Sigma Aldrich), and the resulting hexane-PAH-enriched fractions were filtered through anhydrous sodium sulfate (analytical grade, Honeywell Fluka, Seelze, Germany), and concentrated via a rotary evaporator. The concentrated extract was purified through a silica gel (analytical grade, Merck, Darmstadt, Germany) column (consisting of 20g of 5% water-containing silica gel). The obtained fractions were eluted by 50 mL of hexane/dichloromethane (HPLC grade, Sigma Aldrich), mixture and finally concentrated to 1 ml under a gentle nitrogen gas stream.

#### Extraction of PAHs from water samples

Extraction of PAHs from water samples was undertaken as previously described [26]. Briefly: five hundred milliliters of water sample were transferred into a separating flask, and mixed with 150 ml of hexane, the mixture was vigorously shaken with intervals of pressure release. The mixture was allowed to separate into two distinct upper and lower layers in the flask. The upper organic layer was filtered through a mixture of glass wool and sodium sulfate anhydrous. The same previous steps were repeated another two times. The three extracts were pooled and concentrated overnight in a fume hood. Purification of samples was performed on silica gel column chromatography [27]. Briefly, a silica gel column was prepared with 30 g of activated silica gel (60 mesh), and the column was topped with sodium sulfate anhydrous. Firstly, 20 ml n-hexane was used to raise the prepared column. Secondly, each extracted sample was loaded onto the pre-prepared silica gel column and eluted with 50 ml of n-hexane. The resulting eluates were concentrated under a gentle stream of pure nitrogen gas to 1 ml. Then each concentrated sample was stored at 4°C until GC-MS analysis [28].

#### Determination of the sixteen (16) priority PAHs in all samples

All samples were analyzed for the 16 priority PAHs namely; fluoranthene (Flu), acenaphthylene (Acy), fluorene (Flo), naphthalene (Naph), phenanthrene (Phe), anthracene (Ant), acenaphthene (Ace), o-Terphenyl (O-terph), benzo(a)anthracene (BaA), pyrene (Pyr), chrysene (Chy), benzo(b)fluoranthene (BbF), benzo(k)fluoranthene (BkF), benzo(a)pyrene (BaP), dibenzo(a,h)anthracene (DahA), indeno(1,2,3-cd) pyrene (IcdP), and benzo(g,h, i)perylene (BghiP)) using GC-MS analysis. A mixed standard solution containing the US EPA 16 PAHs was obtained from AccuStandard Inc. (New Haven, CT, USA). The PAH concentrations in the sample extracts were determined by gas chromatography-mass spectrometry with an Agilent 6890N gas chromatograph (GC) interfaced with an Agilent 5973 mass selective detector (Agilent Technologies, Santa Clara, USA).

#### Instrumental analysis

The identification and quantification of individual PAHs were conducted via Gas Chromatography-Tandem Mass Spectrometry (GC-MS/MS) with a DB5 ms ultra-inert capillary column (0.25 mm diameter, 0.25 μm thickness, 30 m length,) for sample separation. The analytical setup comprised a Thermo TRACE^™^ 1300 gas chromatograph with a PTV split mode, injector coupled to a TSQ 8000 Evo mass spectrometer operating in Selected Reaction Monitoring (SRM) mode. The operational parameters included the use of splitless mode with a 1 mL injection volume, employing specific temperature settings and flow rates for carrier gas, transfer, and cleaning processes. Thermos Triplums RSH Autosampler was utilized for sample handling [29]. The quantification relied on the integrated peak-to-area ratio of target ions to external standards, while the identification of PAH analytes was facilitated by specific target ions and retention time sequences. The chromatographic temperature program followed a gradient from 60°C to 300°C over predefined time intervals. Data acquisition, processing, and reporting were executed using Thermo Scientific Excalibur.

Quality assurance measures include daily calibration of standard curves with reference standards and periodic confirmation of calibration levels after each ten analyses. Analyte concentrations in blanks were either negligible or were below the detection limit. The regression coefficient of calibration curves for the TSQ instrument ranged from 0.975 to 0.997. The relative error range of the method precision ranged from 0.1 to 5.0%, which ensures accuracy across the analytical procedures. N-alkanes and PAH residue’s recovery rates of water and sediment samples ranged from 92.4% to 107.8% [30].

The limits of detection (LOD) and quantification (LOQs) were determined as three and ten times the signal-to-noise ratio of the blanks respectively. The LODs and LOQs for the polycyclic hydrocarbons ranged from 0.01 to 0.08 ppb and 0.03 and 0.16ppb.

#### ELCR assessment

ELCR assessment was calculated using an equation adopted from the USEPA, (2004). Detailed calculations are presented in S1 Table.

$$\begin{array}{ll} \text{ELCR} & =\text{CDi}\;\text{x}\;\text{SF}\\ CD_{i} & =\frac{\text{C}\;\text{x}\;\text{IR}\;\text{x}\;\text{EF}\;\text{x}\;\text{ED}}{\text{BW}\;\text{x}\;\text{AT}} \end{array}$$

where:

CDi = Chronic daily intake through ingestion (mg/kg/day).

C = BaP TEQ concentration in water (μg/l)

BaPTEQ = BaPTEF x PAH conc. in water

BaPTEF = BaP relative potency equivalency factor (for BbF, BkF, BaP = 0.11, 0.037 and 1.00 respectively Muller, 1997)

IR = Ingestion rate [children = (1 l/day), adult = (2 l/day).

EF = Exposure frequency (365 days/year).

ED = Exposure time (days/year = 70 years).

BW = Average body weight of the exposed person [for children = 15 kg; adult = 70 kg).

AT = Average time for carcinogens in days (ED x 365 days i.e. 70 x 365 = 25,550 days).

SF = Cancer slope factor (ingestion = 7.3 mg/kg/day^-1^)

Hence, substituting in the equation

ELCR=CDixSF

where:

SF = Cancer slope factor (for ingestion = 7.3 mg/kg/day^-1^)

**For BaF**

$$\begin{array}{ll} \text{ELCR} & =\text{CDi}\;\text{x}\;\text{SF}\\ \text{CDi} & =\frac{0.00033\;\text{x}\;2.5\;\text{x}\;365\;\text{x}\;70\;\text{x}\;10^{-3}}{70\;\text{x}\;25,550\;\text{x}\;\text{7.3}}\\ & =8.61\;x\;10^{-8} \end{array}$$

**For BkF**

$$\begin{array}{ll} \text{ELCR} & =\text{CDi}\;\text{x}\;\text{SF}\\ \text{CDi} & =\frac{0.00011\;\text{x}\;2.5\;\text{x}\;365\;\text{x}\;70\;\text{x}\;10^{-3}}{70\;\text{x}\;25,550\;\text{x}\;\text{7.3}}\\ & =2.87\;x\;10^{-8} \end{array}$$

**For BaP**

$$\begin{array}{ll} \text{ELCR} & =\text{CDi}\;\text{x}\;\text{SF}\\ \text{CDI} & =\frac{0.007\;\text{x}\;2.5\;\text{x}\;365\;\text{x}\;70\;\text{x}\;10^{-3}}{70\;\text{x}\;25,550\;\text{x}\;\text{7.3}}\\ & =1.83\;x\;10^{-6} \end{array}$$

$$\begin{array}{ll} Total & =8.61\;\text{x}\;10^{-8}+2.87\;\text{x}\;10^{-8}+1.83\;\text{x}\;10^{-6}\\ & =1.94\;x\;10^{-6} \end{array}$$

### Data analysis

Data analysis of the results was performed using SPSS (version 23). Student’s t-test, Analysis of Variance (ANOVA), Least Significant Difference (LSD) post hoc, and Pearson’s correlation tests were used to test for mean differences between groups, variations within and among groups, and associations between variables respectively at p ≤ 0.05.

## Results

### PAHs in surface and underground waters in comparison to sachet and bottled water

Results of the 16 USEPA PAHs in underground, surface, sachet, and bottled water samples are shown in Table 1. ANOVA test and in comparison, to WHO and USEP safe limits, our results have shown that non-carcinogenic PAHs (naphthalene, acenaphthylene, acenaphthene, fluoranthene, pyrene) and carcinogenic PAHs (benzo(b)fluoranthene, benzo(k)fluoranthene, and benzo(a)pyrene) were detected in surface water samples; while benzo(b)fluoranthene, benzo(k)fluoranthene, and benzo(a)pyrene were detected in underground water. None of the PAHs were detected in sachet water and bottled water. The PAH levels in both surface and underground water samples were all below the WHO and USEPA limits. The naphthalene, benzo(b)fluoranthene, and benzo(k)fluoranthene levels of surface water samples were significantly higher than the underground water (p = 0.015; 0.047; 0.047 respectively). There were no significant differences in the levels of other PAHs between the 2 water samples. Detailed Polycyclic aromatic hydrocarbons molecular formula and structure is presented in S2 Table.

**Table 1:** Comparison of mean concentration of PAH in underground, surface, sachet, and bottled water samples (ANOVA), and compared to WHO and USEP safe limits.

PAH	Surface Water n = 4	Underground Water n = 6	Satchet Water n = 7	Bottled Water n = 7	F ratio	P value	WHO limits (μg/l)	USEPA Limits (μg/l)
**Non-carcinogenic**								
**Naph (μg/l)**	0.013±0.005	0.00±0.00	ND	ND	40.000	0.000*	30	NA
**Acy (μg/l)**	0.02±0.00	ND	ND	ND	ND	ND	NA	60
**Ace (μg/l)**	0.015±0.007	ND	ND	ND	ND	ND	NA	60
**Flo (μg/l)**	ND	ND	ND	ND	ND	ND	300	NA
**Ant (μg/l)**	ND	ND	ND	ND	ND	ND	2000	NA
**Phe (μg/l)**	ND	ND	ND	ND	ND	ND	NA	60
**Flu (μg/l)**	0.00±0.00	ND	ND	ND	ND	ND	300	NA
**Pyr (μg/l)**	0.007±0.01	ND	ND	ND	0.250	0.667	200	NA
**Carcinogenic**								
**BaA (μg/l)**	ND	ND	ND	ND	ND	ND	NA	NA
**Chy (μg/l)**	ND	ND	ND	ND	ND	ND	NA	0.2
**BbF (μg/l)**	0.02±0.006	0.003±0.006	ND	ND	8.000	0.047*	NA	0.1
**BkF (μg/l)**	0.02±0.006	0.003±0.006	ND	ND	8.000	0.047*	NA	0.2
**BaP (μg/l)**	0.03±0.04	0.007±0.006	ND	ND	1.208	0.334	NA	0.2
**DahA (μg/l)**	ND	ND	ND	ND	ND	ND	NA	0.4
**IcdP (μg/l)**	ND	ND	ND	ND	ND	ND	0.7	0.2
**BghiP (μg/l)**	ND	ND	ND	ND	ND	ND	NA	0.3

Mean±SD; ND = Not Detected; NA = Not Available.

### Detection of PAHs in vegetable, and palm oil samples

Table 2 shows the comparison of the PAH content of vegetable oil and palm oil samples (t-test) and their levels relative to the EU maximum levels. The 16 USEPA priority PAHs were all detected in both palm oil and vegetable oil with the highest level of anthracene in vegetable oil (0.29μg/l) (S1 Fig). The benzo(a)pyrene level was within the maximum limits established by the EU. The anthracene and chrysene levels of vegetable oils were significantly higher than palm oil (p = 0.024; 0.032 respectively). There were no significant differences in the levels of other PAHs between the 2 oil samples.

**Table 2:** Comparison of mean concentration of polycyclic aromatic hydrocarbon in vegetable oil, and palm oil samples (t-test), and compared to EU maximum levels.

PAH	Vegetable oil n = 9	Palm oil n = 3	p-value	EU max. level (μg/kg)
**Non-carcinogenic**				
**Naph (μg/l)**	0.05±0.08	0.02±0.01	0.301	NA
**Acy (μg/l)**	0.05±0.11	0.01±0.01	0.358	NA
**Ace (μg/l)**	0.03±0.01	0.02±0.01	0.197	NA
**Flo (μg/l)**	0.28±0.37	0.19±0.24	0.699	NA
**Ant (μg/l)**	0.29±0.24	0.05±0.07	0.024*	NA
**Phe (μg/l)**	0.06±0.10	0.24±0.37	0.501	NA
**Flu (μg/l)**	0.03±0.03	0.01±0.01	0.182	NA
**Pyr (μg/l)**	0.05±0.10	0.01±0.01	0.259	NA
**Carcinogenic**				
**BaA (μg/l)**	0.05±0.04	0.02±0.01	0.092	NA
**Chy (μg/l)**	0.03±0.02	0.02±0.00	0.032*	NA
**BbF (μg/l)**	0.09±0.09	0.03±0.02	0.087	NA
**BkF (μg/l)**	0.09±0.07	0.05±0.04	0.266	NA
**BaP (μg/l)**	0.12±0.09	0.09±0.01	0.306	2.0
**DahA (μg/l)**	0.12±0.08	0.17±0.02	0.159	NA
**IcdP (μg/l)**	0.11±0.08	0.13±0.00	0.799	NA
**BghiP (μg/l)**	0.10±0.09	0.06±0.01	0.159	NA

Mean±SD; ND = Not Detected; NA = Not Available.

Table 3 shows a comparison of the mean concentration of non-carcinogenic polycyclic aromatic hydrocarbon in barbecued food and fresh food (FF) samples using analysis of variance. Significant variations were observed in the levels of naphthalene, acenaphthylene, acenaphthene, fluorene, phenanthrene, anthracene, fluoranthene, and pyrene among all the barbecued food and their fresh equivalent food samples studied (p < 0.05). No significant difference was observed in the benzo(a)anthracene levels of all samples (p > 0.05).

**Table 3:** Comparison of mean concentration of non-carcinogenic PAHs in barbecued food and fresh food (FF) samples.

PAH	BBQ Beef	BBQ Fish	BBQ Plan	BBQ Pork	BBQ Yam	BBQ Chic	BBQ Chev	BBQ Pota	BBQ Corn	FF Beef	FF Fish	FF Plant	FF Pork	FF Yam	FF Chic	FF Chev	FF Pota	FF Corn	F ratio	P value
**Naph (μg/l)**	0.02±0.0009	0.01±0.0009	0.05±0.001	0.09±0.09	0.03±0.002	0.01±0.0004	0.04±0.02	0.06±0.03	0.23±0.08	0.01±0.00	0.02±0.00045	0.09±01	0.03±0.001	0.01±0.0004	0.01±0.00	0.03±0.02	0.01±0.01	0.02±0.01	14.193	0.000*
**Acy (μg/l)**	0.009±0.005	0.0002±0.004	0.03±0.0009	0.02±0.0004	0.04±0.0009	0.01±0.0009	1.04±0.09	0.01±0.00	0.37±0.11	0.01±0.0009	0.009±0.004	0.00±0.00	0.00±0.00	0.00002±0.00005	0.0002±0.0004	0.03±0.02	0.01±0.00	ND	232.122	0.000*
**Ace (μg/l)**	0.0002±0.004	ND	0.000±0.0005	ND	ND	ND	1.03±0.11	0.03±0.02	0.54±0.21	ND	ND	ND	ND	ND	ND	0.05±0.02	0.01±0.00	ND	75.567	0.000*
**Flo (μg/l)**	0.008±0.004	ND	0.01±0.002	ND	ND	ND	1.02±0.12	0.01±0.00	0.06±0.02	ND	ND	ND	ND	ND	ND	0.01±0.01	0.02±0.00	ND	221.777	0.000*
**Ant (μg/l)**	0.02±0.0008	0.01±0.0004	0.02±0.001	0.05±0.07	ND	0.01±0.0009	0.05±0.02	0.07±0.00	0.20±0.13	ND	ND	ND	ND	ND	ND	0.01±0.00	ND	ND	5.446	0.000*
**Phe (μg/l)**	0.02±0.007	0.01±0.007	0.02±0.002	0.01±0.001	ND	0.01±0.0008	1.64±0.17	0.01±0.00	1.28±0.34	ND	ND	ND	ND	ND	ND	0.06±0.04	ND	0.01±0.00	110.633	0.000*
**Flu (μg/l)**	0.00±0.00	ND	0.00002±0.00005	0.0002±0.0004	ND	ND	0.03±0.02	0.01±0.00	0.99±0.14	ND	ND	ND	ND	ND	ND	0.02±0.02	ND	ND	185.586	0.000*
**Pyr (μg/l)**	0.00002±0.00005	0.0000±0.00005	0.0002±0.0005	0.00002±0.00005	ND	0.00002±0.00005	0.03±0.02	0.01±0.00	0.06±0.03	ND	ND	ND	ND	ND	ND	0.02±0.00	ND	ND	12.50	0.000*

Mean±SD; ND = Not Detected; NA = Not Available.

Additionally, the comparison of the mean concentration of non-carcinogenic polycyclic aromatic hydrocarbon levels of barbecued food and FF samples using LSD post hoc is shown in Table 4 & S2 Fig. The naphthalene levels of barbequed pork, potato, and corn were significantly higher than those of fresh pork, potato, and corn respectively (p = 0.002, 0.017, <0.001). The levels of other PAHs were not significantly different between barbequed and FF samples (p>0.05).

**Table 4:** Comparison of the mean concentration of non-carcinogenic polycyclic aromatic hydrocarbon levels of barbecued food and FF samples using LSD post hoc.

PAH	Groups		Mean diff	P value
	Barbecue Pork	Fresh Pork		
**Naph (μg/l)**	0.09±0.101	0.03±0.001	0.062±0.018	0.002*
	Barbecue Potato	Fresh potato		
	0.06±0.03	0.01±0.01	0.048±0.018	0.017*
	Barbecue Corn	Fresh Corn		
	0.23±0.08	0.02±0.01	0.21±0.018	<0.001*

On the other hand, the mean concentration of carcinogenic PAH in barbecued food and FF samples using analysis of variance is presented in Table 5. A significant difference was observed in the levels of chrysene, benzo(b)fluoranthene, benzo(k)fluoranthene, benzo(a)pyrene, indeno(1,2,3-cd)pyrene, dibenzo(a,h)anthracene, and benzo(g,h, i)perylene) among all the barbecued and their fresh equivalent food samples studied (p<0.05). However, no significant differences were observed in the levels of carcinogenic PAHs between individual barbequed food sample and their corresponding fresh equivalent ((p>0.05).

**Table 5:** Comparison of the mean concentration of carcinogenic PAH levels in barbecued food and fresh food (FF) samples.

PAH	BB Beef	BB Fish	BB Plan	BB Pork	BB Yam	BB Chic	BB Chev	BB Pota	BB Corn	FF Beef	FF Fish	FF Plant	FF Pork	FF Yam	FF Chic	FF Chev	FF Pota	FF Corn	F ratio	P value
**BaA (μg/l)**	ND	ND	ND	ND	ND	ND	0.03±0.02	0.02±0.00	0.27±0.19	ND	ND	ND	ND	ND	ND	0.06±0.00	0.01±0.00	ND	2.178	0.174
**Chy (μg/l)**	ND	ND	ND	ND	ND	ND	0.06±0.02	0.02±0.00	0.31±0.12	ND	ND	ND	ND	ND	ND	0.03±0.00	0.02±0.00	ND	7.466	0.012*
**BbF (μg/l)**	0.00002±0.00005	0.009±0.005	0.00002±0.00005	0.01±0.0004	ND	0.01±0.0004	0.17±0.07	0.03±0.00	0.61±0.13	ND	0.01±0.0009	ND	ND	ND	ND	0.11±0.00	0.02±0.00	ND	56.739	0.000*
**BkF (μg/l)**	0.00002±0.0004	0.01±0.0009	0.00002±0.00005	0.01±0.0009	ND	0.01±0.0009	0.32±0.11	0.08±0.00	0.89±0.20	ND	0.01±0.0009	ND	ND	ND	ND	ND	0.01±0.00	ND	60.664	0.000*
**BaP (μg/l)**	0.00002±0.00005	0.006±0.005	0.0002±0.00004	0.03±0.001	ND	0.02±0.0009	0.27±0.36	0.07±0.00	0.82±0.19	ND	0.01±0.001	ND	ND	ND	ND	0.17±0.00	ND	ND	13.559	0.000*
**DahA (μg/l)**	ND	0.00±0.00	ND	ND	ND	0.01±0.0009	0.13±0.04	0.19±0.00	0.29±0.10	ND	ND	ND	ND	ND	ND	0.23±0.00	ND	ND	21.419	0.000*
**IcdP (μg/l)**	ND	ND	ND	ND	ND	ND	0.11±0.05	0.16±0.06	0.68±0.09	ND	ND	ND	ND	ND	ND	0.30±0.00	0.01±0.00	ND	79.991	0.000*
**BghiP (μg/l)**	ND	0.01±0.0004	ND	ND	ND	0.01±0.0004	0.07±0.03	0.03±0.00	0.28±0.09	ND	ND	ND	ND	ND	ND	0.20 ±0.00	0.01±0.00	NA	24.892	0.000*

Mean±SD; ND = Not Detected; NA = Not Available.

### Detection of PAHs in fresh vegetable samples

As can be seen in Table 6 and S3 Fig the mean concentration of the 16 USEPA priority PAHs in fresh vegetables were all detected in bitter leaf with naphthalene having the highest concentration (0.74μg/kg). The sum of PAHs in fresh vegetable samples was 2.63μg/kg while the sum of the carcinogenic PAHs (PAH8) was 0.65 μg/kg accounting for 24.7% of the total PAHs in all the vegetable samples studied.

**Table 6:** Mean concentration of PAHs in fresh vegetables.

PAHs Vegetables	Naph	Acy	Ace	Flo	Ant	Phe	Flu	Pyr	BaA	Chy	BbF	BkF	BaP	DahA	IcdP	BghiP
	(μg/kg)	(μg/kg)	(μg/kg)	(μg/kg)	(μg/kg)	(μg/kg)	(μg/kg)	(μg/kg)	(μg/kg)	(μg/kg)	(μg/kg)	(μg/kg)	(μg/kg)	(μg/kg)	(μg/kg)	(μg/kg)
**Water leaf**	ND	0.01	0.02	0.01	0.01	ND	ND	ND	ND	ND	ND	ND	ND	ND	ND	0.01
**Bitter leaf**	0.74	0.01	0.01	0.21	0.03	0.01	0.01	0.01	0.01	0.02	0.07	0.10	0.06	0.10	0.22	0.03
**Cabbage**	0.02	0.01	0.01	0.03	0.03	0.04	ND	ND	ND	0.01	ND	ND	ND	0.01	ND	ND
**Carrot**	0.03	0.01	0.02	0.01	0.02	0.01	ND	ND	ND	0.01	ND	ND	ND	ND	ND	ND
**Cucumber**	0.02	ND	ND	ND	0.04	0.02	ND	ND	ND	ND	ND	ND	ND	ND	ND	ND
**Pumpkin**	ND	0.01	ND	0.10	0.03	ND	ND	ND	ND	ND	ND	ND	ND	ND	ND	ND
**Garlic**	0.01	ND	ND	0.01	ND	0.04	ND	ND	ND	ND	ND	ND	ND	ND	ND	ND
**Ginger**	0.03	ND	ND	ND	0.02	ND	ND	ND	ND	ND	ND	ND	ND	ND	ND	ND
**Green leaf**	0.03	0.04	ND	ND	ND	0.10	ND	ND	ND	ND	ND	ND	ND	ND	ND	ND
**Okazi**	0.02	ND	ND	0.02	ND	ND	ND	ND	ND	ND	ND	ND	ND	ND	0.05	ND
**Onion**	0.07	0.04	0.01	ND	ND	ND	ND	ND	ND	ND	ND	ND	ND	ND	ND	ND
**Pepper**	ND	ND	ND	ND	ND	ND	ND	ND	ND	ND	0.02	0.05	0.04	0.03	ND	ND

Mean±SD; ND = Not Detected; NA = Not Available.

### Health Risk assessment of carcinogenic PAHs to residents of the study area

The sum of carcinogenic and non-carcinogenic PAHs in FF, barbequed food, vegetables, cooking oil, and water samples is shown in Table 7 and S4 Fig. The order of concentrations of total PAHs in samples was barbequed food > cooking oil > FF > fresh vegetables > water while that for carcinogenic PAHs was barbequed food > cooking oil > fresh vegetables > FF > water. The highest concentration of total PAHs was seen in barbequed food (67.71 μg/kg) while the lowest concentration was seen in water (0.40 μg/kg). The highest concentration of carcinogenic PAHs was also seen in barbequed food (24.40 μg/kg) while the lowest concentration was seen in water (0.26 μg/kg). PAH8 levels were higher than the EU maximum value in PAH4 (12.0 μg/kg) for PAHs in smoked meat and meat products. The sum of carcinogenic PAH levels in cooking oil was below the EU maximum value Table 7.

**Table 7:** Sum of carcinogenic and non-carcinogenic PAHs in fresh food, barbequed food, vegetables, cooking oil, and water samples.

Sample	Carcinogenic PAHs (μg/kg)	Non-carcinogenic PAHs (μg/kg)	Total PAHs (μg/kg)	EU Maximum level PAH4(μg/kg)
**Fresh Food**	**0.95**	**1.81**	**2.76**	**NA**
**Barbequed food**	**24.40**	**43.31**	**67.71**	**12**
**Vegetables**	**0.65**	**1.98**	**2.63**	**NA**
**Cooking Oil**	**7.14**	**8.80**	**15.94**	**10**
**Water Samples**	**0.26**	**0.14**	**0.40**	**NA**

PAH4 = Sum of benzo(a)pyrene, benz(a)anthracene, benzo(b)fluoranthene and chrysene

The Benzo(a)pyrene toxic equivalent quotients, BaP relative potency equivalency factors, and Estimated lifetime cancer risk for residents of the study area are shown in Table 8. The estimated lifetime cancer risk accruing from exposure to carcinogenic PAHs by ingestion is within the WHO and USEPA safe limits. The residents of the study area are relatively safe from cancer risk from ingestion of food, water, and vegetables in the study area.

**Table 8:** Benzo(a)pyrene toxic equivalent quotients, BaP relative potency equivalency factors and estimated lifetime cancer risk for residents of the study area.

Carcinogenic PAHs	BaP TEF	Conc. in water	BaP TEQ	Calculated ELCR	WHO ELCR	USEPA ELCR
**BbF (μg/l)**	**0.11**	**0.003**	**0.00033**	**8.61 x 10** ^**−8**^		
**BkF (μg/l)**	**0.037**	**0.003**	**0.00011**	**2.87 x 10** ^**−8**^		
**BaP (μg/l)**	**1.0**	**0.007**	**0.007**	**1.83 x 10** ^**−6**^		
**Total**			**0.00744**	**1.94 x 10** ^**−6**^	**8.7 x 10** ^**−5**^	**1 x 10** ^**−6**^ **–1 x 10** ^**−4**^

BaP TEQ = (BaP) toxic equivalent quotients; BaP TEF = BaP relative potency equivalency factors adapted from Muller, (1997).

## Discussion

Concerns about the current environmental PAH status stem from their persistent, toxic, carcinogenic and distribution within different ecosystems. Additionally, their incorporation into the food chain raised a significant threat to the human health [31] with nearly 30% of human cancer cases linked to low-level PAH exposure through dietary intake [17]. For the above-presented concerns, this study focused on evaluating the PAH content in various sources such as surface and underground water samples, vegetable oils, palm oils, fresh vegetables, barbecued foods, and their fresh equivalents. Additionally, the study assessed ELCR associated with the ingestion of PAH-contaminated foods in Nigeria.

Our study showed that the PAH content of all water, oil, vegetable, and food samples studied was within the USEPA/WHO safe limits. This observation can be attributed to the low industrial and traffic density of the study area. PAHs are slowly biodegradable under aerobic conditions; thus, PAH contamination of food and water is, therefore, a function of the environmental concentration of PAH [32]. Contrary to our observation, PAH levels exceeding the maximum permissible limit recommended by WHO for drinking water, groundwater, and aquatic environment have been reported by previous reports [14, 33, 34]. Additionally, PAH levels exceeding the EU safe limits for vegetable oils have also been reported [35]. The mean PAH levels recorded in this study (0.001–0.02 μg/L; Table 1) were lower compared to levels (46–507 μg/L) reported by [13] who analyzed water samples around Atlas Cove, Lagos, Nigeria; and lower than the reports from [14] (119 ± 204 μg L−1) who worked on groundwater in Ife North local government area of Osun state, Nigeria; were also lower compared to the values of (11.2–341.5μg L−1) reported by [36]. Higher levels of PAHs observed in these areas compared to our study area may be attributed to the dense population, heavy traffic, heavy industrialization, and the associated environmental pollution that characterizes these areas.

The naphthalene, benzo(b)fluoranthene, and benzo(k)fluoranthene levels of surface water samples (0.013±0.005; 0.02±0.0006; 0.02±0.0006) were significantly higher than the underground water samples (0.000±0.00; 0.003±0.006; 0.003±0.006). Our observation is consistent with those of [37], who also reported that the total PAH concentrations were significantly higher in surface water than in groundwater (0.80 μg/L, 0.46 μg/L) regardless of seasons and higher carcinogenic PAHs concentrations in surface water than those in groundwater only in the summer season. This implies the health risk associated with drinking PAH-contaminated surface water. Higher PAH levels in surface water than underground water may be attributed due to large surface area being exposed to PAH contamination from different anthropogenic activities (pyrolysis (burning) of organic matter (waste or food), motor vehicle exhaust, petroleum refineries, oil/gasoline spills, tobacco smoke, barbeque smoke, and coke production [2]. Contrary to our finding, a previous work demonstrated higher PAH contamination of groundwater samples which they attributed to different industrial activities in these areas [38].

The PAH levels in fresh vegetables were within the range of 0.001 to 0.8 μg/kg. The highest PAH occurrence is observed in bitter leaf which demonstrated all the 16 PAHs studied. Naphthalene was detected in all vegetables except water leaf and fluted pumpkin. The remaining PAHs were not detected in significant concentration in all studied vegetables. Relative high amounts of low-molecular-weight PAHs such as naphthalene, acenaphthylene, and acenaphthene have been detected in the majority of vegetables and fruits [39]. Variations in PAH accumulation in a variety of vegetables have been reported by previous studies [19, 40]. The PAH concentration difference in different vegetables can be attributed to species-specific characteristics, not the soil or the environmental conditions, as all of the samplings share common soil and environmental conditions. In plants, a proportional correlation is found between leaves surface area and PAH concentration due to the increase of PAH absorption from the atmosphere [41]. A previous study confirmed that leafy vegetables such as spinach, white gourds, fenugreeks, chilis, ribbed gourds, and cauliflowers were more contaminated with PAHs compared to potatoes, radishes, and turnips which are underground vegetables [19]. Furthermore, waxy surface vegetables can accumulate particle-bound HMW-PAHs via atmospheric deposition and LMW-PAHs via surface adsorption [42]. On the other hand, it has been reported that vegetables possessing large and ragged surface leaves such as cauliflowers, cabbages, and grapes have the power of particulate matter trapping which contributes to the increase in the concentration of four-ring PAHs [17, 43]. The waxy and large surface area of the leaves of the bitter leaf may be responsible for the presence of all the studied PAHs observed in the bitter leaf. Although studies have shown that PAHs are found at low concentrations in fresh vegetables, the lipophilic and high biological half-lives properties of PAHs allow them to bioaccumulate and concentrate in human tissues via dietary consumption of contaminated crops [44].

The levels of all PAHs in both vegetable oil and palm oil (Table 2) were within the USEPA and WHO safe limits. A similar observation has been made by a previous study [45]. Contrary to our findings, the BaP content of edible vegetable oils has been shown to exceed the EU standard limits of (2 μg/kg). The PAH4 in edible oils (including palm oil and peanut oil) exceeding the EU maximum limit of 10 μg/kg has also been reported [46]. The vegetable oils in this study had higher anthracene and chrysene levels (Table 2; 0.29±0.24 μg/kg; 0.03±0.02 μg/kg) compared to palm oil (0.05±0.07μg/kg; 0.02±0.00 μg/kg). Higher PAH levels have also been reported in retail vegetable oil with a Benzo(a)pyrene level of 1.2 μg/kg while palm oil has been shown to have the lowest PAH concentrations [47]. Contrary to our findings, Palm oil has been shown to emit significantly higher particle-phase PAHs than soybean oil and olive oil. Cyclopenta(c,d)pyrene was the predominant particle-phase PAH, accounting for 62, 56, and 37% of the particle-phase PAH for soybean oil, palm oil, and olive oil, respectively [48]. Higher PAH levels have been demonstrated in palm oil compared to peanut oil samples in Northern Nigeria [46]. Higher levels of PAH in vegetable oils can be attributed to different factors such as their lipophilic characteristics, the utilization of hot air (from firing process) to dry oily seeds (which increase PAHs deposition in seeds), and the deposition of PAHs in oily seeds from soil, air, and water and during packaging processes [49].

The levels of naphthalene, acenaphthylene, Phenanthrene, benzo(b)fluoranthene, benzo(k)fluoranthene, benzo(a)pyrene, and dibenzo(a,h)anthracene varied significantly among the barbecued and FF samples studied (Table 3). Consistent with our findings, it was reported that differences in PAH concentration were observed according to meat and PAH type. Additionally, they showed that dietary intake of PAH is a function of PAH-contaminated food consumption level [50]. Barbecued pork, potato, and corn recorded significantly higher levels of naphthalene (Table 4; 0.09±0.101 μg/kg; 0.06±0.03μg/kg; 0.23±0.08μg/kg) compared to their fresh equivalents (0.03±0.001μg/kg; 0.01±0.01μg/kg; 0.02±0.01μg/kg). Such observations were not made for other barbequed food studies with their fresh equivalents. The roasting process has been shown to lead to an increase in PAH content in plant foods. PAH levels reportedly observed for raw *Zea mays* (0.6 mg kg-1 d.w.) may reach 6.9 mg kg-1 d.w when roasted over a charcoal fire [51]. A report from a previous study recorded that no PAH was detected in yam fresh and potato fresh (LB = 0). The boiling and/or pounding of yam and potato did not generate any PAH15 + 1 congener above the analytical limit [46].

The PAH levels in fresh fish, chicken, yam, and plantain chevon were not significantly different from their barbecued equivalent. Contrary to our findings, samples of charcoal-grilled chicken and fish were reported to contain the highest level of ΣPAHs (9.46 μg/kg). PAHs formed in grilled meat over a charcoal fire are dependent on meat fat content, and the duration and temperature of the cooking [52]. Furthermore, PAH concentration in different tissues is dependent on tissue fat content [1]. Grilling meat with appreciable fat content would contribute to a higher accumulation of PAHs while; in contrast, food with low fat content contains an insignificant amount of PAHs [53]. Therefore, the high-fat content of pork may be responsible for the higher PAH levels observed in barbecued pork meat compared to raw meat.

The highest concentration of total PAHs and carcinogenic PAHs were observed in barbequed food (67.71 μg/kg; Table 7) while the lowest concentrations were observed in water (0.40 μg/kg; Table 7). Consistent with our findings, previous studies have reported that some of the highest concentrations of PAHs have been found in food cooked over open flames. Grilled Salmon and beef samples have been shown to display the highest PAH concentrations [54]. Alomirah’s group evaluated the concentrations and profiles of 16 PAHs in various grilled and smoked foods and detected eight genotoxic PAHs; among these, Chr (4.88 μg/kg) and BaA (2.27 μg/kg) had the highest mean values [55]. The genotoxic PAH8 and total PAHs (ƩPAHs) were reported to record the highest average levels (3.09 and 36μg/kg) respectively, in charcoal grilled chicken samples [56]. Grilled foods as a common food in home and markets represent a health risk to the population due to higher concentrations of carcinogenic PAH in comparison to foods prepared by alternative cooking methods [57]. The total PAH levels of 0.82 μg/kg lower than our values (2.63 μg/kg; Table 7) have been demonstrated in vegetables by [58]. The total PAHs in water samples studied (0.40 μg/kg; Table 7) were lower than values obtained in water samples in Woji (1.3935 mg/L) and Eleme in (3.009 mg/L) in Southern Nigeria. Total levels of carcinogenic PAHs ranging from 0.0014 to 0.9429 mg/L have been reported in borehole water samples [59].

Additionally, the study assessed the estimated lifetime cancer risk (ELCR) associated with the ingestion of PAH-contaminated foods. our study showed that the PAH content of all water, oil, vegetable and food samples studied were within the USEPA/WHO safe limits. The ELCR risk from this present study (1.94 x 10^−6^) was within the WHO (8.7 x 10^−5^) and USEPA (1 x 10–6–1 x 10^−4^) safe limits. In consonance with our findings, the average upper-bound carcinogenic risks associated with the consumption of water both from rivers and urban water supplies were reported to be less than 1 x 10^−4^ (Pan et al, 2015). A study on the PAH content of locally consumed vegetables and the risk assessment of ΣPAH4 also reported that there was no risk associated with the consumption of the vegetable samples [60]. found that PAH contamination in fried potatoes and similar products does not pose a direct threat to the health of consumers [61].

## Study limitation and future research plan

Limitations of the study include the low number of samples, especially water samples, and the need to extend the research to other geographical areas. Future research would focus on the assessment of PAH metabolite titer in human subjects residing nearby of the collection site.

## Conclusion

Our results have shown that surface water contains significantly higher PAHs than in borehole samples (*naphthalene*, *benzo(b)fluoranthene*, *and benzo(k)fluoranthene*) while vegetable oils had higher anthracene and chrysene. Barbecued pork, potato, and corn had significantly higher PAHs than other samples collected in this study Consumption of barbecued food and surface water may be associated with higher exposure to polycyclic aromatic hydrocarbons which may predispose to increased risk of chronic health conditions in the absence of any preventive and remediation measures.

## Supporting information

S1 Table Estimation of lifetime Cancer Risk (ELCR). (DOCX)

S2 Table Polycyclic aromatic hydrocarbons molecular formula and structure. (DOCX)

S1 Fig Comparison of the mean concentration of polycyclic aromatic hydrocarbon in vegetable and palm oil samples (t-test) and compared to EU maximum levels. (PPTX)

S2 Fig Comparison of mean concentrations of non-carcinogenic polycyclic aromatic hydrocarbon in barbecued food and fresh food samples using LSD post hoc. (PPTX)

S3 Fig Polycyclic aromatic hydrocarbon content of different vegetable samples. (PPTX)

S4 Fig Sum of carcinogenic and non-carcinogenic PAHs in fresh food, barbequed food, vegetables, cooking oil, and water samples. (PPTX)
