# Supplementary material for: Polycyclic aromatic hydrocarbons content of food, water and vegetables and associated cancer risk assessment in Southern Nigeria
Source: PLoS One. 2024 Jul 23;19(7):e0306418. doi: 10.1371/journal.pone.0306418 (PMC11265677; doi:10.1371/journal.pone.0306418)
Supplement: S1 Table — (DOCX) [file pone.0306418.s001.docx]

**S1 Table: Estimation of lifetime Cancer Risk (ELCR)**

ELCR assessment was calculated using an equation adopted from the USEPA, (2004).

Estimated Lifetime Cancer Risk (ELCR) = CDi x SF

**CD_i_**  = C x IR x EF x ED

BW x AT

where:

CDi = Chronic daily intake through ingestion (mg/kg/day).

C = BaP TEQ concentration in water (µg/l)

BaPTEQ = BaPTEF x PAH conc. in water

BaPTEF = BaP relative potency equivalency factor (for BbF, BkF, BaP = 0.11, 0.037 and

1.00 respectively Muller, 1997)

IR = Ingestion rate [children = (1 l/day), adult = (2 l/day).

EF = Exposure frequency (365 days/year).

ED = Exposure time (days/year = 70 years).

BW = Average body weight of the exposed person [for children = 15 kg; adult = 70 kg).

AT = Average time for carcinogens in days (ED x 365 days i.e. 70 x 365 = 25,550 days).

SF = Cancer slope factor (ingestion= 7.3 mg/kg/day^-1^)

Hence, substituting in the equation **ELCR = CDi x SF**

where:

SF = Cancer slope factor (for ingestion= 7.3 mg/kg/day^-1^)

**For BaF**

ELCR= CDi x SF

CDi = 0.00033 x 2.5 x 365 x 70 x 10^-3^

70 x 25,550 x 7.3

= 21.07875 x 10^-3^

1,788,500

= 0.0000118 x 10^-3^ = 1.18 x 10^-8^

= 1.18 x 10^-8^ x 7.3

= **8.61 x 10^-8^**

**For BkF**

ELCR = CDi x SF

CDi = 0.00011 x 2.5 x 365 x 70 x 10^-3^

70 x 25,550 x 7.3

= 7.02625 x 10^-3^

1,788,500

= 0.00000393 x 10^-3^ = 3.39 x 10^-9^

=0.00000393 x 10^-3^ x 7.3

= **2.87 x 10^-8^**

**For BaP**

ELCR= CDi x SF

CDI= 0.007 x 2.5 x 365 x 70 x 10^-3^

70 x 25,550 x 7.3

= 447.125 x 10^-3^

1,788,500

= 0.00025 x 10^-3^ = 3.39 x 10^-9^

= 0.00025 x 10^-3^ x 7.3 = **1.83 x 10^-6^**

**Total** = 8.61 x 10^-8^ **+** 2.87 x 10^-8^ + 1.83 x 10^-6^

= **1.94 x 10^-6^**
