# Supplementary material for: Polycyclic aromatic hydrocarbons content of food, water and vegetables and associated cancer risk assessment in Southern Nigeria
Source: PLoS One. 2024 Jul 23;19(7):e0306418. doi: 10.1371/journal.pone.0306418 (PMC11265677; doi:10.1371/journal.pone.0306418)
Supplement: S2 Table — (DOCX) [file pone.0306418.s002.docx]

**Supplementary Table 2** Polycyclic aromatic hydrocarbons molecular formula and structure.

| **PAHs Congeners** | **Molecular**  **formula** | **Structure** |
| --- | --- | --- |
| Naphthalene (Nap) | C_10_H_8_ | 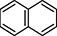 |
| Acenaphthylene (Acy) | C_12_H_8_ | 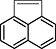 |
| Acenaphthene (Ace) | C_12_H_8_ | 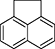 |
| Fluorene (Flu) | C_13_H_10_ | 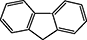 |
| Anthracene (Ant) | C_14_H_10_ | 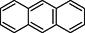 |
| Phenanthrene (Phe) | C_14_H_10_ | 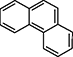 |
| Fluoranthene (Flt) | C_16_H_10_ | 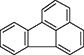 |
| Pyrene (Pyr) | C_16_H_10_ | 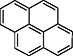 |
| Cyclopenta[*c,d*]pyrene (CPP) * | C_18_H_10_ | 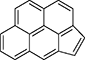 |
| Benzo[*a*]anthracene (BaA) * | C_18_H_12_ | 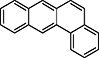 |
| Chrysene (Chry) * | C_18_H_12_ | 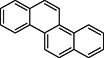 |
| 5-Methylchrysene (5MeCh) * | C_19_H_14_ | 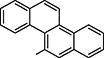 |
| Benzo[*b*]fluoranthene (BbF) * | C_20_H_12_ | 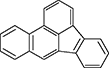 |
| Benzo[*k*]fluoranthene (BkF) * | C_20_H_12_ | 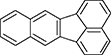 |
| Benzo[*j*]fluoranthene (BjF) * | C_20_H_12_ | 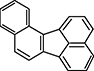 |
| Benzo[*a*]pyrene (BaP) * | C_20_H_12_ | 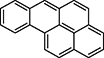 |
| Indeno[*1,2,3-cd*]pyrene (IP) * | C_22_H_12_ | 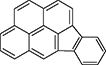 |
| Benzo[*g,h,i*]perylene (BghiP) * | C_22_H_12_ | 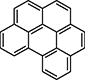 |
| Dibenzo[*a,h*]anthracene (DBahA) * | C_22_H_14_ | 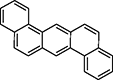 |
| Dibenzo[*a,l*]pyrene (DBalP) * | C_24_H_14_ | 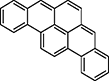 |
| Dibenzo[*a,e*]pyrene (DBaeP) * | C_24_H_14_ | 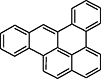 |
| Dibenzo[*a,i*]pyrene (DBaiP) * | C_24_H_14_ | 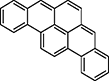 |
| Dibenzo[*a,h*]pyrene (DBahP) * | C_24_H_14_ | 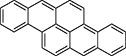 |
