# Supplementary material for: Polycyclic aromatic hydrocarbons content of food, water and vegetables and associated cancer risk assessment in Southern Nigeria
Source: PLoS One. 2024 Jul 23;19(7):e0306418. doi: 10.1371/journal.pone.0306418 (PMC11265677; doi:10.1371/journal.pone.0306418)
Supplement: S1 Fig — (PPTX) [file pone.0306418.s003.pptx]

## Slide 1
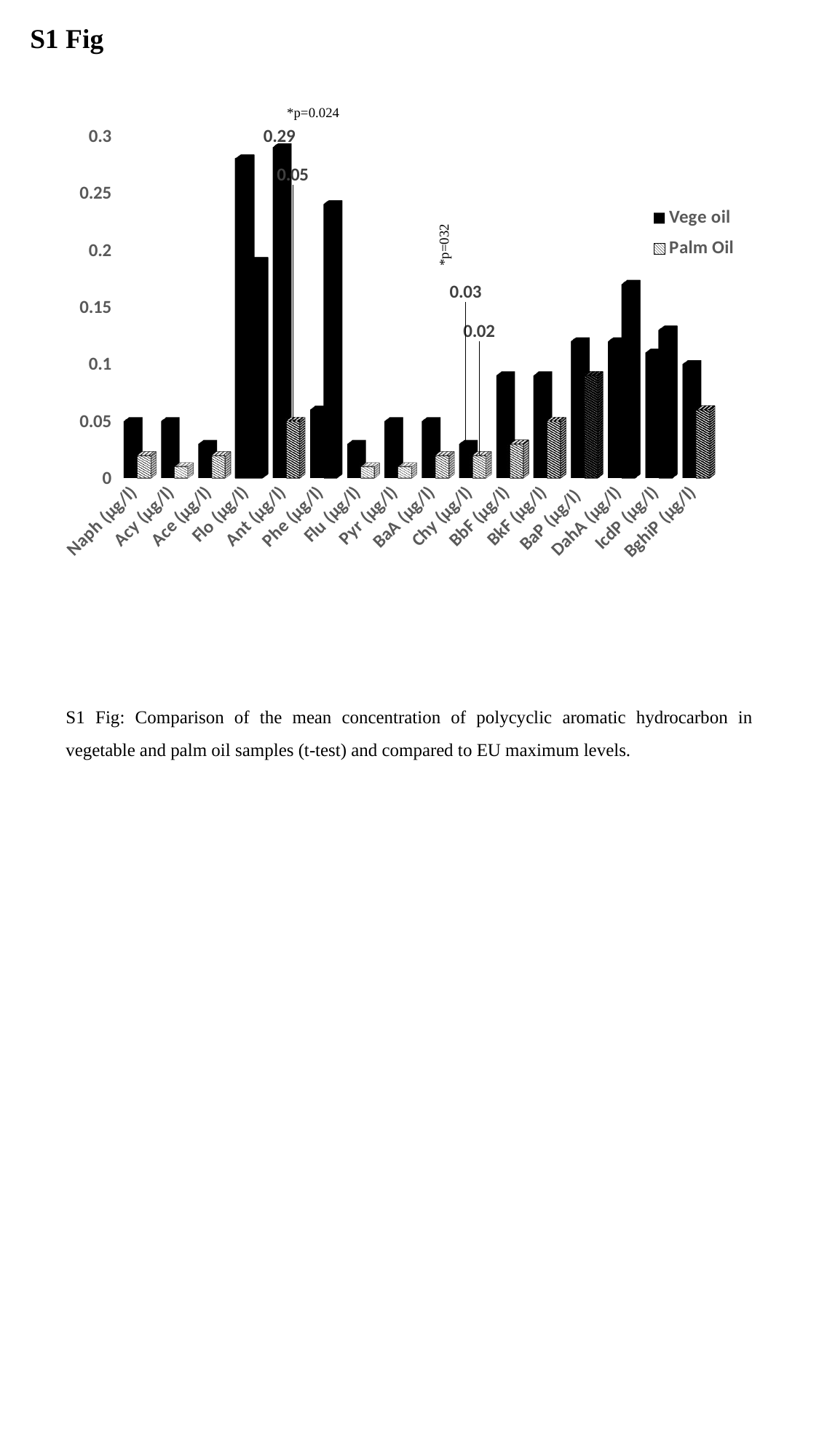

S1 Fig
[unsupported chart]
S1 Fig: Comparison of the mean concentration of polycyclic aromatic hydrocarbon in vegetable and palm oil samples (t-test) and compared to EU maximum levels.
