# Supplementary material for: Polycyclic aromatic hydrocarbons content of food, water and vegetables and associated cancer risk assessment in Southern Nigeria
Source: PLoS One. 2024 Jul 23;19(7):e0306418. doi: 10.1371/journal.pone.0306418 (PMC11265677; doi:10.1371/journal.pone.0306418)
Supplement: S2 Fig — (PPTX) [file pone.0306418.s004.pptx]

## Slide 1
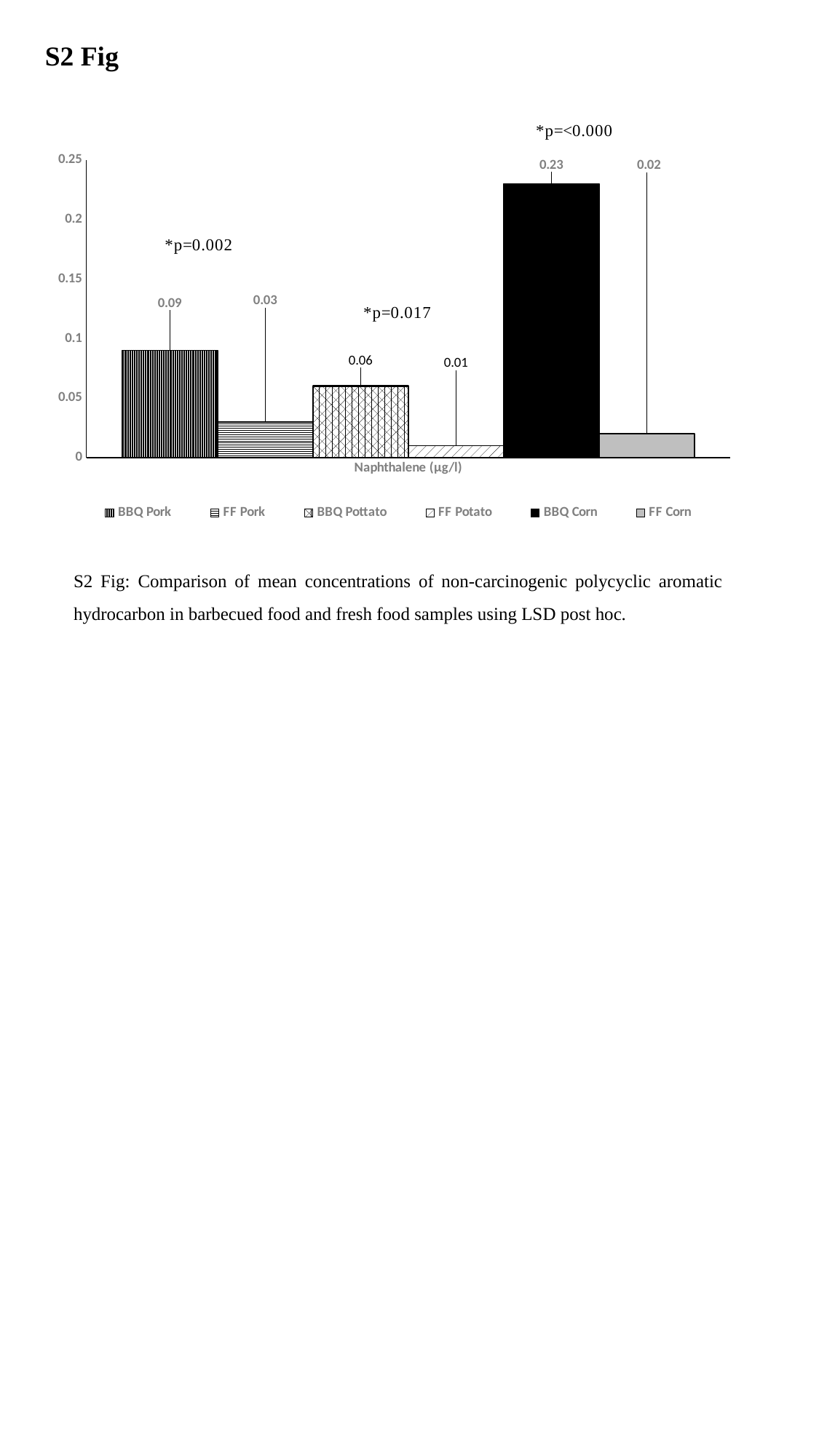

S2 Fig
### Chart
| Category | BBQ Pork | FF Pork | BBQ Pottato | FF Potato | BBQ Corn | FF Corn |
|---|---|---|---|---|---|---|
| Naphthalene (µg/l) | 0.09 | 0.03 | 0.06 | 0.01 | 0.23 | 0.02 |S2 Fig: Comparison of mean concentrations of non-carcinogenic polycyclic aromatic hydrocarbon in barbecued food and fresh food samples using LSD post hoc.
