# Supplementary material for: Polycyclic aromatic hydrocarbons content of food, water and vegetables and associated cancer risk assessment in Southern Nigeria
Source: PLoS One. 2024 Jul 23;19(7):e0306418. doi: 10.1371/journal.pone.0306418 (PMC11265677; doi:10.1371/journal.pone.0306418)
Supplement: S3 Fig — (PPTX) [file pone.0306418.s005.pptx]

## Slide 1
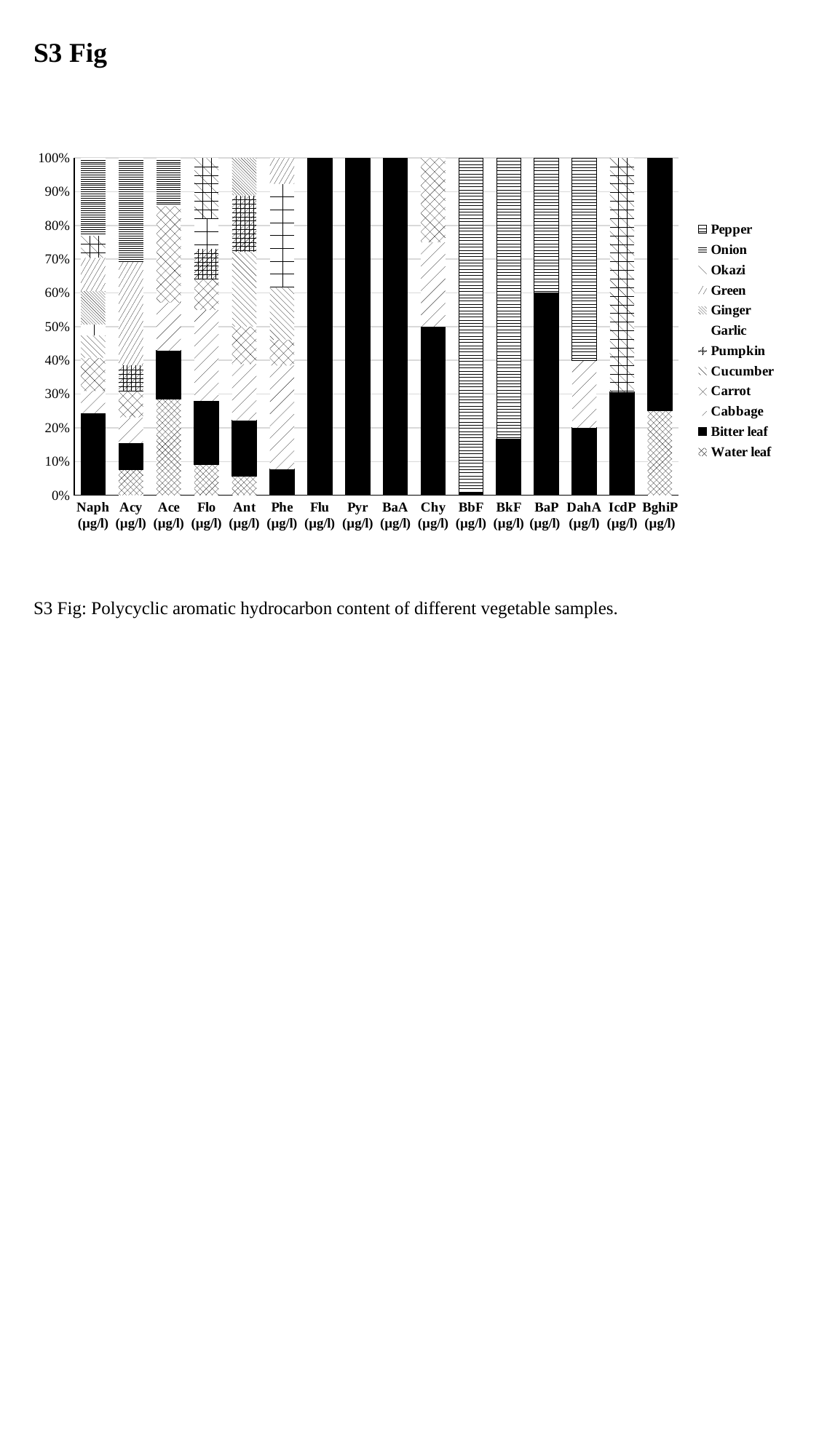

S3 Fig
### Chart
| Category | Water leaf | Bitter leaf | Cabbage | Carrot | Cucumber | Pumpkin | Garlic | Ginger | Green | Okazi | Onion | Pepper |
|---|---|---|---|---|---|---|---|---|---|---|---|---|
| Naph (µg/l) | 0.0 | 7.4 | 2.0 | 3.0 | 2.0 | 0.0 | 1.0 | 3.0 | 3.0 | 2.0 | 7.0 | 0.0 |
| Acy (µg/l) | 1.0 | 1.0 | 1.0 | 1.0 | 0.0 | 1.0 | 0.0 | 0.0 | 4.0 | 0.0 | 4.0 | 0.0 |
| Ace (µg/l) | 2.0 | 1.0 | 1.0 | 2.0 | 0.0 | 0.0 | 0.0 | 0.0 | 0.0 | 0.0 | 1.0 | 0.0 |
| Flo (µg/l) | 1.0 | 2.1 | 3.0 | 1.0 | 0.0 | 1.0 | 1.0 | 0.0 | 0.0 | 2.0 | 0.0 | 0.0 |
| Ant (µg/l) | 1.0 | 3.0 | 3.0 | 2.0 | 4.0 | 3.0 | 0.0 | 2.0 | 0.0 | 0.0 | 0.0 | 0.0 |
| Phe (µg/l) | 0.0 | 1.0 | 4.0 | 1.0 | 2.0 | 0.0 | 4.0 | 0.0 | 1.0 | 0.0 | 0.0 | 0.0 |
| Flu (µg/l) | 0.0 | 1.0 | 0.0 | 0.0 | 0.0 | 0.0 | 0.0 | 0.0 | 0.0 | 0.0 | 0.0 | 0.0 |
| Pyr (µg/l) | 0.0 | 1.0 | 0.0 | 0.0 | 0.0 | 0.0 | 0.0 | 0.0 | 0.0 | 0.0 | 0.0 | 0.0 |
| BaA (µg/l) | 0.0 | 1.0 | 0.0 | 0.0 | 0.0 | 0.0 | 0.0 | 0.0 | 0.0 | 0.0 | 0.0 | 0.0 |
| Chy (µg/l) | 0.0 | 2.0 | 1.0 | 1.0 | 0.0 | 0.0 | 0.0 | 0.0 | 0.0 | 0.0 | 0.0 | 0.0 |
| BbF (µg/l) | 0.0 | 0.07 | 0.0 | 0.0 | 0.0 | 0.0 | 0.0 | 0.0 | 0.0 | 0.0 | 0.0 | 7.0 |
| BkF (µg/l) | 0.0 | 1.0 | 0.0 | 0.0 | 0.0 | 0.0 | 0.0 | 0.0 | 0.0 | 0.0 | 0.0 | 5.0 |
| BaP (µg/l) | 0.0 | 6.0 | 0.0 | 0.0 | 0.0 | 0.0 | 0.0 | 0.0 | 0.0 | 0.0 | 0.0 | 4.0 |
| DahA (µg/l) | 0.0 | 1.0 | 1.0 | 0.0 | 0.0 | 0.0 | 0.0 | 0.0 | 0.0 | 0.0 | 0.0 | 3.0 |
| IcdP (µg/l) | 0.0 | 2.2 | 0.0 | 0.0 | 0.0 | 0.0 | 0.0 | 0.0 | 0.0 | 5.0 | 0.0 | 0.0 |
| BghiP (µg/l) | 1.0 | 3.0 | 0.0 | 0.0 | 0.0 | 0.0 | 0.0 | 0.0 | 0.0 | 0.0 | 0.0 | 0.0 |S3 Fig: Polycyclic aromatic hydrocarbon content of different vegetable samples.
