# Supplementary material for: Polycyclic aromatic hydrocarbons content of food, water and vegetables and associated cancer risk assessment in Southern Nigeria
Source: PLoS One. 2024 Jul 23;19(7):e0306418. doi: 10.1371/journal.pone.0306418 (PMC11265677; doi:10.1371/journal.pone.0306418)
Supplement: S4 Fig — (PPTX) [file pone.0306418.s006.pptx]

## Slide 1
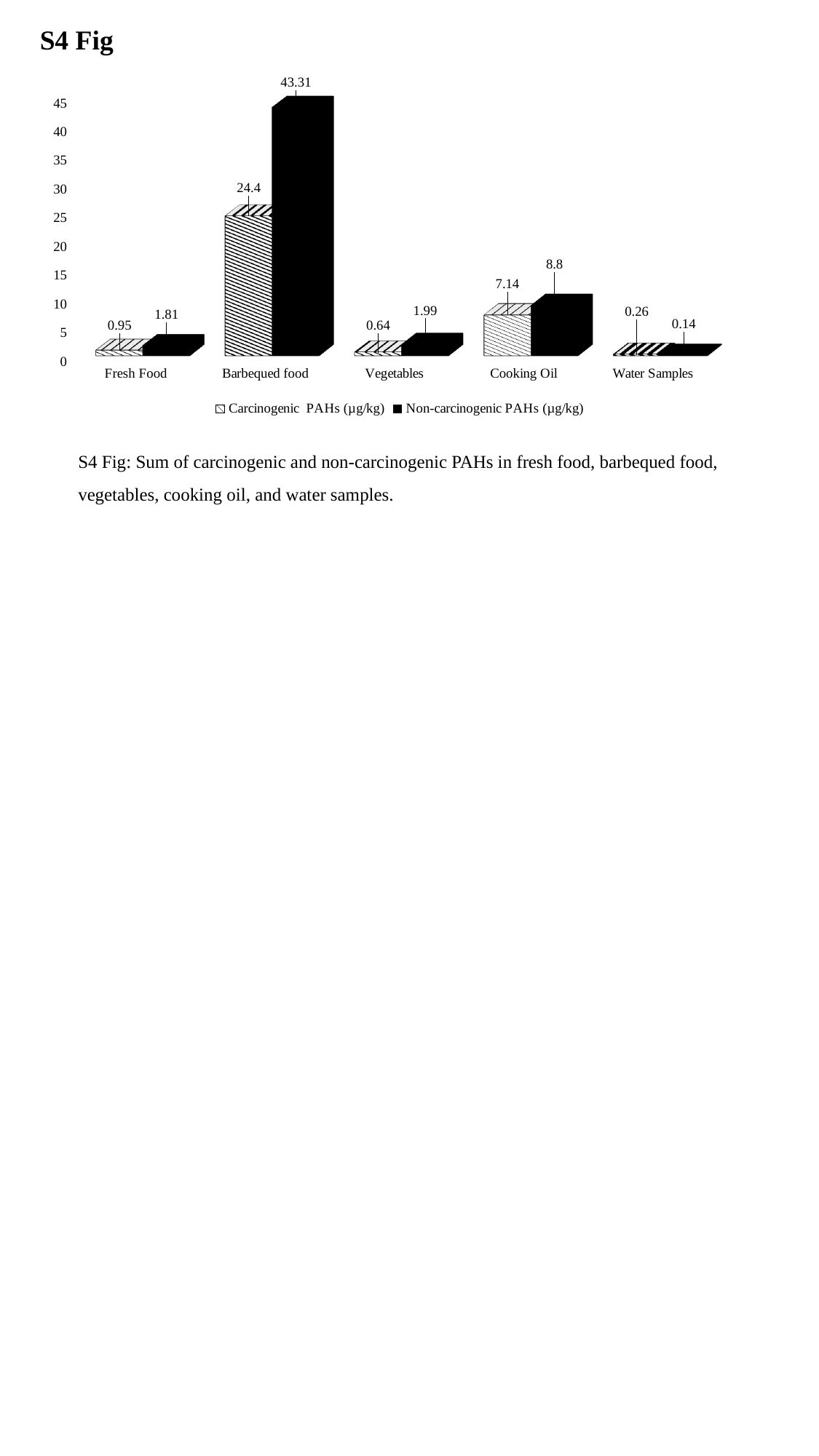

S4 Fig
[unsupported chart]
S4 Fig: Sum of carcinogenic and non-carcinogenic PAHs in fresh food, barbequed food, vegetables, cooking oil, and water samples.
